# Effect of Adherence to Mediterranean Diet during Pregnancy on Children’s Health: A Systematic Review

**DOI:** 10.3390/nu11050997

**Published:** 2019-05-01

**Authors:** Carlotta Biagi, Mattia Di Nunzio, Alessandra Bordoni, Davide Gori, Marcello Lanari

**Affiliations:** 1 Pediatric Emergency Unit, Department of Medical and Surgical Sciences (DIMEC), St. Orsola Hospital, University of Bologna, Via Massarenti 11, 40138 Bologna (BO), Italy; marcello.lanari@unibo.it; 2 Department of Agri-Food Sciences and Technologies (DISTAL), University of Bologna, Piazza Goidanich 60, 47521 Cesena, Italy; mattia.dinunzio@unibo.it (M.D.N.); alessandra.bordoni@unibo.it (A.B.); 3 Interdepartimental Center for Agro-Food Industrial Research (CIRI-AGRO), University of Bologna, via Quinto Bucci 336, 47521 Cesena (FC), Italy; alessandra.bordoni@unibo.it; 4 Department of Biomedical and Neuromotor Sciences, University of Bologna, 40125 Bologna, Italy; davide.gori4@unibo.it

**Keywords:** Mediterranean diet, pregnancy, offspring, child health

## Abstract

The traditional Mediterranean diet has been shown to be a healthy eating pattern that protects against the development of many diseases in adults and children. Pregnancy is a critical period of plasticity during which foetal development may be significantly influenced by different environmental factors, including maternal nutrition. In this context, several studies have examined the potential benefits of adherence to a Mediterranean diet during pregnancy on birth outcomes, considering the Mediterranean diet as a whole rather than focusing on the effect of its individual components. In this review, we systematically summarized and discussed results of studies investigating the protective role of Mediterranean diet against foetal growth, prematurity, neural tube defects and other congenital pathologies, asthma and allergy, body weight and metabolic markers. Although current data are insufficient and randomized control trials are needed, growing evidence suggests the beneficial effect of the Mediterranean diet during pregnancy on children’s health. In this sense, strategies aiming to promote adherence to this dietary pattern might be of considerable importance to public health.

## 1. Introduction

The Developmental Origins of Health and Disease (DOHaD) hypothesis posits that in utero exposure plays a critical role in the risk of disease in adulthood. Maternal diet during pregnancy contributes to the in-utero environment [1]; nutritional stress/stimulus applied during critical periods of early development permanently influences organism’s physiology and metabolism, with the consequences of this metabolic programming often being observed much later in life [2,3]. Although the DOHaD hypothesis is well documented in animals [4], evidence connecting maternal diet quality during pregnancy and offspring risk factors is scarce and inconsistent in humans. Most studies have investigated the associations of specific nutrients, foods, or food groups intake during pregnancy with offspring health without considering the overall diet [5,6].

Foetal growth restriction (FGR) and risk of new-borns small for gestational age (SGA), prematurity, neural tube defects (NTDs), congenital heart defects (CHDs), gastroschisis, asthma and allergy, overweight and metabolic disorders represent leading causes of childhood diseases that are supposed to be connected to maternal nutrition.

FGR is defined as an estimated foetal weight or abdominal circumference below the 5th or the 10th centile according to gestational age (GA) and sex, and it affects about 5–10% of all pregnancies [7]. FGR is associated with an increased risk for childhood morbidity (mainly hypoglycaemia, developmental delay and infectious diseases) and with about half of all foetal deaths [8,9]. It is also associated with chronic diseases in adult life including coronary heart disease, stroke, type-2 diabetes mellitus, adiposity, metabolic syndrome and osteoporosis [10,11]. New-borns are considered SGA when their body weight is lower than the 10th centile according to neonatal growth curves adjusted for gestational age at delivery and sex [12]. Not all SGA new-borns are pathologically growth restricted, as a proportion of babies (18–22%) are constitutionally small but healthy [12]. However, FGR significantly overlaps SGA, and they are often considered as a single entity. FGR and SGA can arise from several maternal, foetal and placental problems [13]; however, maternal nutrition has been recognized as one of the most important environmental factors influencing foetal growth and development [14,15,16,17].

Preterm birth is defined as any birth before 37 weeks of GA [18]. Preterm birth, especially before 34 weeks of GA, is the leading cause of perinatal morbidity and mortality in developed country [19]. There are multiple risk factors for premature birth including having a previous premature birth, pregnancy with multiple babies, infection, drug or alcohol use, and age. A well-balanced diet during pregnancy has been reported to reduce the odds of a premature birth [20].

NTDs are a major health burden that affect 0.5–2/1000 pregnancies worldwide and represent a preventable cause of stillbirth, infant death and significant lifelong morbidity [21]. CHDs and gastroschisis are other common malformations representing major causes of mortality, morbidity and disability of perinatal origin [22,23]. The aetiology of these congenital malformations is multifactorial and both genetic predisposition and environmental influences contribute to them, with nutritional deficits serving as potential contributing factors [24,25].

The prevalence of asthma and allergic diseases (atopic dermatitis/eczema, allergic rhino-conjunctivitis) has increased worldwide over the past few decades with the highest incidence occurring in children [26]. Globalization and consequent deviation from traditional to Western diet might be one of the environmental changes involved in the recent increased of the atopic diseases. In fact, decreased antioxidant (fruit and vegetables), increased n-6 polyunsaturated fatty acids (PUFA) (margarine, vegetable oil), and decreased n-3 PUFA (fish oil) intakes might lead to oxidative stress and inflammation and might contribute to the higher incidence of asthma and allergies [27,28].

Due to the importance of maternal diet during pregnancy as influencer of child health, actions to improve its quality are urgently needed. In this light, a good adherence to the Mediterranean diet (MD) could represent a good strategy. MD is characterized by increased consumption of unprocessed and plant foods, olive oil, and fish, whereas consumption of red meat, animal fats, sugars and salt are minimal. The MD is rich in mono-unsaturated fatty acids (MUFA), omega-3 PUFA and antioxidant polyphenols, and it is been recommended for its overall health benefits and potential for disease prevention [29]. Several observational and intervention studies support the role of the MD in preventing obesity, type 2 diabetes mellitus and metabolic syndrome in adults [30,31], while some recent studies suggest a protective role against obesity development in children [32,33,34]. In pregnancy, a higher adherence to the MD has been associated with lower risk of preterm birth, and higher birth weight [20].

The aim of this systematic review is to verify the association between maternal adherence to the MD during pregnancy and children health outcomes, and to provide clinicians with levels and quality of evidence on the efficacy of MD in improving paediatric health outcomes.

## 2. Methods

### 2.1. Study Selection

This systematic review was performed according to the Preferred Reporting Items for Systematic Reviews and Meta-analyses guidelines (PRISMA) [35].

We searched electronic databases (September 17, 2018) Medline, EMBASE and Clinical Trials. The search process was conducted using the following keywords: (pregnancy OR gravidity OR pregnant OR pregnant women OR pregnan*) OR (child OR children OR childhood OR paediatric OR paediatric OR paediatric* OR paediatric OR offspring OR new-born OR new-borns OR neonate OR neonates Or neonatal OR toddler OR toddlers) AND (“Diet, Mediterranean” (Mesh) OR Mediterranean diet* OR Med Diet OR Mediterranean diet). Inclusion criteria were: (i) English language; (ii) systematic recording of diet during the gestational period (daily register or food frequency questionnaire—FFQ) in healthy women; (iii) assessment of “small for gestational age”, prematurity, foetal growth, neural tube defects, asthma, wheeze, atopy, insulin resistance and metabolic syndrome, childhood overweight, metabolic markers, epigenetic and congenital pathologies in the offspring.

Exclusion criteria were: (i) irrelevant titles not indicating the research topic; (ii) evaluation of dietary patterns different from the MD; (iii) dietary intervention including single/few nutrients or single/few aspects of dietary intake; (iv) dietary intervention with inadequate description of the dietary treatment. There was no restriction regarding period or publication status.

**Figure 1:**
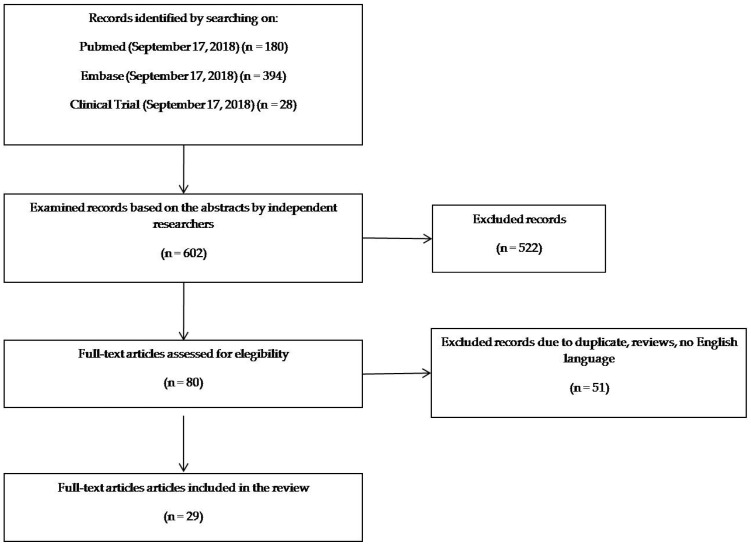
diagram of search strategy and study selection.

Titles and abstracts of studies initially identified from databases (602 studies) were checked by two independent investigators (C.B. and M.D.N.) and disagreements among reviewers were resolved through a mediator (M.L.). After first screening, duplicates, reviews, letters, abstracts and articles without full-text in English language were also excluded. The detailed selection process is presented in Figure 1.

### 2.2. Study Quality Assessment

Two researchers (C.B. and M.D.N.) independently and blindly assessed the risk of bias of included studies using the parameters defined by the Cochrane Tool for Quality Assessment [36] and the Strengthening the Reporting of Observational Studies in Epidemiology (STROBE) [37]. Disagreement was resolved primarily through discussion and consensus between the researchers. If consensus was not reached, another blind reviewer (D.G) acted as third arbiter.

The Cochrane Tool analyses seven bias groups: sequence generation and allocation concealment (both within the domain of selection bias or allocation bias), blinding of participants and personnel (performance bias), blinding of outcome assessors (detection bias), incomplete outcome data (attrition bias), selective reporting (reporting bias) and an auxiliary domain: “other bias”. For each bias group, it is possible to assign a value of “high,” “low” or “unclear” risk of bias when it is not specified if a specific bias is present or not. Every bias judgment helps to assign a global assessment to every RCT according to the Agency for Healthcare Research and Quality (AHRQ) standards (good, fair and poor).

The STROBE statement is a 22 items tool specifically designed to evaluate observational studies quality. Items are associated with the different sections of an article, such as title and abstract (item 1), introduction (items 2 and 3), methods (items 4–12), results (items 13–17), discussion (items 18–21), and other information (item 22 on funding). Eighteen items are identical for the three different study designs, while four (specifically items 6, 12, 14, and 15) are differentially designed for each study type (i.e., cohort or case control). STROBE does not provide a scoring stratification. As a rule, the higher the score, the higher the quality of the study. We hence created three score thresholds corresponding to three levels of items scored: 0–14 as poor quality, 15–25 as intermediate quality and 26–33 as good quality of the study.

## 3. Results

### 3.1. Search Findings

The initial search identified 602 studies, with 522 records excluded following abstract review (Figure 1). Of the 80 articles retrieved, 51 were excluded because of duplicates, reviews, letters, abstracts and articles without full-text in English language. In the end, 29 studies were included in the analysis. Included studies were published between 2008 and 2018.

### 3.2. Studies Characteristics

A detailed description of the selected articles is reported in Table 1.

Risk of FGR and of SGA new-borns were the outcomes addressed by the higher number of studies (8 out of 29 studies), followed by asthma and allergy in the offspring (7 out of 29), and prematurity risk (6 out of 29). Two studies [41,49] recurred in 2 different outcomes, FGR and prematurity, owing to multiple comparison groups between the articles. All studies except [49] were observational studies: 16 cohort studies, 5 case-control and 7 cross sectional studies.

### 3.3. Risk of bias and Quality of Reporting

We conducted the quality analysis based on the aforementioned methods and tools for analysis. Table 2 shows the main quality results scored by the papers included. Of the 29 articles included for quality analysis, 16 (55%) were cohort studies, 5 (17%) were case-control studies and 7 (24%) were cross sectional studies. One paper (3%) had an RCT study design. Six cohort studies (38%) were of good quality, 7 (44%) of intermediate quality and 3 (18%) of poor quality. All the 5 case control studies were of intermediate quality. Among cross sectional studies, five (71%) were of intermediate quality and 2 (29%) of poor quality.

### 3.4. Evidence Synthesis

Results coming from the 29 selected articles are reported below according to the main outcome of the study.

#### 3.4.1. Foetal Growth Restriction and Small for Gestational Age

Evidence for this outcome comes from 8 papers (Table 1, Table 2): an RCT of poor quality, 4 cohort studies (1 good, 3 intermediate quality), 2 cross sectional studies (1 intermediate and 1 poor quality) and 1 case-control study of intermediate quality. Cohort studies reached the best scores for quality.

In few studies, adherence to MD was assessed in early pregnancy since the trajectory of foetal growth is set at this stage [11]. The Generation R prospective observational study [38] evaluated the association between dietary patterns in the early phase of pregnancy (GA < 18 weeks) and foetal size/weight at birth in 3207 pregnant women living in Rotterdam. To test their adherence to MD over the preceding three months, a semiquantitative FFQ including 293 food items was self-administered at enrolment (median GA = 13.5 weeks). Women were categorised into tertiles of adherence to the MD (low, medium and high) based on their intake of vegetables, vegetable oil, fish, fruits, pasta and rice, meat, potatoes and fatty sauces. Low adherence to the MD in early pregnancy resulted associated with decreased intra-uterine size and lower birth weight compared to high adherence (difference in grams at birth −72 [95% CI: −110.8 to −33.3]).

Chatzi et al. [39] analysed the impact of MD adherence (MDA) during the first trimester of pregnancy on foetal growth in the Spanish multicentre INMA cohort (2461 mother-new-born pairs), divided into the Atlantic area and the Mediterranean area cohort, and during the mid-trimester in the Greek RHEA cohort (889 mother-new-born pairs). Semi-quantitative FFQ respectively were administered by trained interviewers. Maternal MDA was evaluated through a score including 100 or 250 food items modified from the MD score by Trichopoulou [40]. The modified score was specifically designed for pregnant women, and it considered dairy food protective and did not include alcohol consumption. Food intake and MD score differed significantly across cohorts, mean MD score being higher in the INMA-Mediterranean and RHEA cohorts compared to the INMA-Atlantic cohort. Women with high MD adherence had a significantly lower risk of delivering an FGR infant (OR 0.5 [95% CI: 0.3–0.9]) only in the INMA-Mediterranean cohort. Of note, in all cohorts, high MD adherence increased child weight at birth in smoking mothers, thus suggesting a counteraction of the detrimental effect of smoking. In most studies, adherence to the MD was evaluated in the last trimester of pregnancy or after delivery. Saunders and colleagues [41] found no association between maternal adherence to MD and the risk of FGR in 728 pregnant women enrolled in Guadeloupe. A semi-quantitative FFQ including 214 food items was administered by trained interviewers in the days following delivery, and adherence to the MD was evaluated using the Trichopoulou 9-level score [40].

In a Spanish cross-sectional prospective study [42], a semi-quantitative FFQ comprising 127 food items was administered by trained interviewers to 127 women (46 and 81 mothers with SGA and appropriate for gestational age foetuses, respectively) during the third trimester of pregnancy. Adherence to the MD was calculated according to Trichopoulou [40]. A good adherence appeared a protective factor for SGA, with an OR 0.18 (95% CI: 0.74–0.501) for the third consumer quartile.

An association between low maternal MDA and risk of SGA in new-borns was evidenced in another Spanish retrospective, cross-sectional population-based study involving 492 pregnant women [43]. A 16 items semi-quantitative FFQ was self-administered after delivery, and adherence to the MD was evaluated according to a modified version of the KidMed score [44] considering optimal (>7 score) and low (<7 score) adherence. Women with low MD adherence had a higher risk of delivery SGA babies (adjOR 1.68 [95% CI: 1.02–5.46) when adjusting for parental body mass index (BMI) and multiple gestation, but not when adjusting for all significant possible confounders.

The single-centre, prospective, observational cohort study by Parlapani et al. [45] evaluated the relation between MD adherence and size at birth in 82 pregnant women who delivered preterm singletons (post-conceptional age <34 weeks). MD adherence was calculated according to the Dietary Score of Panagiotakos et al. [46] based on a self-administered semi-quantitative FFQ (156 food items). Neonates from mothers in the high-MD adherence group were less likely to be SGA (OR 3.3 [95% CI: 1.24–8.78]).

Interestingly, a recent multi-centre, matched case-control, Spanish study [47] evaluated the effect of MD adherence and olive oil intake during pregnancy on the risk of SGA infants using three different scores: PREDIMED score [48], Trichopoulou’ s score [40] and Panagiotakos’ score [46]. Five hundred eighteen mothers of SGA infants and 518 mothers of infants with normal weight for GA were enrolled, and a137-items semi-quantitative FFQ was administered by trained interviewers within two days after delivery. Independent of the score, adherence to the MD and daily consumption of 5 gr of olive oil was associated to a reduced risk of SGA in the new-born (adjOR 0.59 [95% CI: 0.38–0.98]).

To date, only one intervention study correlating adherence to the MD and SGA has been published [49]. The primary aim of this Spanish, randomized, controlled trial was the evaluation of the incidence of gestational diabetes mellitus (GDM) in pregnant women at 8–12 weeks GA. Five hundred women were randomly assigned to intervention group (MD supplemented with extra virgin olive oil and pistachios) or control group (standard diet with limited fat intake); secondary neonatal outcomes included SGA and prematurity (<37 GA). MD adherence was assessed according to the Mediterranean Diet Adherence Screener (MEDAS) score [50] based on a semi-quantitative 14-items FFQ administered by trained interviewers during the 4 study visits (first ultrasound visit, 24–28 GA, 36–38 GA, delivery). According to this score, women in the interventional group had a good adherence to the intervention. A significant reduction of SGA (p 0.001) was observed in the intervention arm.

#### 3.4.2. Prematurity

Evidence for this outcome comes from 6 papers (Table 1, Table 2): an RCT of poor quality, 4 cohort studies (1 good, 3 intermediate quality) and 1 cross sectional study of poor quality. Cohort studies reached the best scores for quality.

MDA was not significantly associated with the risk of preterm delivery in 5738 American women who delivered non-malformed infants and participated as controls in the National Birth Defects Prevention Study, a multicentre, population-based, case-control study conducted in the United States [51]. MD adherence in the year before pregnancy was evaluated 6 weeks-24 months after delivery administering by telephone interview the computer-based semi-quantitative 58-item FFQ developed in the Nurses’ Health Study [79]. The MD Trichopoulou’s score [40] and the Diet Quality Index (DQI) incorporating pregnancy-specific nutritional recommendations [52] were calculated and they were not associated with preterm delivery. Notwithstanding, results should be interpreted with caution due to the poor quality of this study and the very low overall incidence of early preterm delivery (about 1%). Moreover, the study examined dietary habits during the year before pregnancy and subsequent substantial changes were not considered.

Saunders et al. [41] reported no overall associations between MD adherence during pregnancy and the risk of preterm delivery in a French Caribbean population having a dietary pattern similar to MD. However, a decreased risk was reported in overweight and obese woman (adjOR 0.7 [95%CI: 0.6–0.9).

In 2008, adherence to the MD during pregnancy was reported to be associated with reduced risk of early preterm birth (<35 weeks of GA) in Denmark [53] but not in Norway [55]. In the Danish prospective cohort studies 35657 women [53] received a semi-quantitative FFQ (360-item) by mail in mid-pregnancy (gestation week 25). The questionnaire covered the diet during the previous four weeks. In the Norwegian cohort (26563 women) a semi-quantitative FFQ (255 items) investigating dietary habits before pregnancy was self-administered at week 18–22 of gestation [55]. For both studies, adherence to MD was assessed based on 5 major criteria defined by Khoury [54]: consumption of fish twice a week or more, 5 or more vegetable/fruit servings per day, use olive or rapeseed oil, meat at most twice a week, and no more than 2 cups of coffee per day. High MD adherence was associated with reduced the risk of early preterm birth (adjOR 0.28 [95% CI: 0.11–0.76]) in the Danish cohort. In both studies no association emerged regarding the risk of late preterm delivery (35–36 weeks of GA).

An association between MD and preterm delivery was also evidenced by Smith and colleagues [56], who analysed the associations between late and moderately preterm (LMPT) birth (32–37 weeks of GA) and maternal lifestyle factors (smoking, alcohol, drug use and diet) in a population-based case-cohort study involving the mothers of 922 LMPT and 965 term singletons born in UK. Lifestyle and dietary information during pregnancy were obtained via maternal interview shortly after delivery, and women were considered adherent to MD if their diet included at least 1 of the following: 5 portions of fruits and vegetables every day, fish more than twice a week, meat less than twice a week, maximum of 2 cup of coffee a day. Although not adherent women (2.6%) were almost twice as likely to deliver LMPT as adherent women (RR 1.81 [95% CI: 1.04–3.14]), it is worth considering that the “not adherent” diet was very poor, and the effect could be related to the highly unbalanced dietary pattern.

To date, the already mentioned randomized, controlled, trial by Assaf-Balut [49] is the only available intervention study on this topic. It demonstrated that an early nutritional intervention based on MD supplemented with extra virgin olive oil and pistachios significantly reduces the rate of prematurity (p 0.023).

#### 3.4.3. Neural Tube Defects

Evidence for this outcome comes from 2 cross sectional studies of intermediate quality (Table 1, Table 2).

The case-control study by Vujkovic et al. involved 50 mothers of children with spina bifida and 81 control mothers [57]. Validated semi-quantitative FFQ (200 food items) were filled out 14 months after delivery covering the nutrient intake 3 months before the study moment. All FFQ were individually checked for consistency at the hospital or by telephone by the researchers. Principal component factor analysis (PCA) and reduce rank regression (RRR) were used to identify a comparable dietary pattern, which was labelled Mediterranean as it was characterised by high intake of vegetables, fruits, vegetable oils, legumes, fish, alcohol and cereal products and low intakes of potatoes and sweets. Low adherence to the MD, according to both PCA and RRR, was associated to a significantly increased risk of spina bifida compared with high adherence (OR 2.7 [95% CI: 1.2–6.1] and OR 3.5 [95% CI: 1.5–7.9]), respectively.

The multi-centre, population-based, case-control study by Carmicheal et al. [58] was conducted in the United States between 1997 and 2005 and it included 936 cases with NTDs, 2475 with orofacial clefts and 6147 controls. Telephone interviews were conducted between 6 weeks and 24 months after delivery to investigate the mother average intake of foods in the year before pregnancy using a semi-quantitative 58-item FFQ. The MD score of Trichopoulou [40], and the DQI incorporating pregnancy-specific nutritional recommendations [52] were calculated. Higher MD score and DQI appeared associated with reduced birth defect risks (adjOR 0.64 [95% CI: 0.45–0.92]), with a stronger association for anencephaly (adjOR 0.49 [95% CI: 0.31–0.75]). Association was still present after adjusting for maternal intake of mineral/vitamin supplements.

#### 3.4.4. Congenital Heart Defects and Gastroschisis

We found two studies assessing the effects of maternal adherence to the MD on two congenital anomalies, CHDs and gastroschisis (Table 1). Both papers had a cross sectional design and scored intermediate quality (Table 2).

In the year 1997–2009, the National Birth Defects Prevention Study [59], a population-based, multicentre, case–control study enrolled 9885 mothers of babies with major CHDs and 9468 mothers of unaffected babies. Maternal interviews administrated 13 months for cases and 9 months for controls after delivery were standardised, computer-based and conducted primarily by telephone in English or Spanish. Interviews included a validated semiquantitative 58-items FFQ focused on consumption in the year before pregnancy. Maternal diet quality was assessed by the DQI for pregnancy [52] and the MD score of Trichopoulou [40]. Quartile 1 (Q1) and 4 (Q4) reflected the worst and best diet quality. An inverse association between better diet quality scores and risk for selected conotruncal and septal defects was present, where the inverse associations were typically weaker for MDS compared with DQI-P. For MDS, a significantly estimated risk reduction (Q4 vs. Q1) was associated only for perimembranous ventricular septal defects (14%, OR 0.86 [95% CI: 0.69–1.07]). For DQI-P, an estimated risks reductions were 37% for tetralogy of Fallot (OR 0.63 [95% CI: 0.49–0.80]), 24% for all conotruncal defects (OR 0.76 [95% CI: 0.64–0.91]), 23% for atrial septal defects (OR 0.77 [95% CI: 0.63–0.94]) and 14% for all septal defects (OR 0.86 [95% CI: 0.75–1.00]).

The National Birth Defects Prevention Study also investigated the relationship between maternal diet quality during the year before conception and gastroschisis in 1125 case mothers and 9483 control mothers (estimated delivery dates between 1997 and 2009) [60]. Diet quality was assessed by DQI [52] and Trichopoulou score [40] based on a validated semiquantitative 58-item FFQ administered as part of the computerized-assisted telephone interview (CATI). A statistically significant decrease of gastroschisis was associated to increasing diet quality for both the DQI and MDS. When stratified by maternal race/ethnicity, this finding was confined to Hispanic women. Among Hispanic women, the risk of gastroschisis decreased significantly with increasing DQI quartiles: quartile 2, aOR 0.58 (95% CI: 0.40–0.86); quartile 3, aOR 0.52 (95% CI: 0.36–0.79); and quartile 4, aOR0.48 (95% CI: 0.32–0.76). Increasing diet quality, as measured by the MDS, showed reduced risk of gastroschisis among women, mostly Hispanic, who were born outside the United States: quartile 2, aOR 0.62 (95% CI: 0.33–1.16); quartile 3, aOR 0.51 (95% CI: 0.28–0.94); and quartile 4, aOR 0.50 (95% CI: 0.28–0.90).

#### 3.4.5. Asthma and Allergy

We retrieved 7 studies exploring the effect of maternal adherence to the MD and incidence of asthma and/or allergic diseases in the offspring (Table 1). Five studies were cohort studies (3 good, 1 intermediate quality and 1 of poor quality) and 2 were cross sectional studies (1 intermediate and 1 poor quality) (Table 2).

Two studies examined the incidence of allergic diseases in the offspring at 6.5 years of age. In the cross-sectional study by Chatzi et al. [61], involving 460 Spanish children, a semi-quantitative 42-item FFQ was administered to mothers three months after delivery by a face-to-face interview to investigate their dietary habits during pregnancy. Children dietary pattern at 6.5 years was evaluated using a semi-quantitative 96-item FFQ administered to parents by an interviewer. MD adherence was evaluated using the Trichopoulou score [40]. A high MDS during pregnancy resulted to be protective for persistent wheeze (adjOR 0.22 [95% CI: 0.08–0.58]), atopic wheeze (adjOR 0.30 [95% CI: 0.1–0.9]) and atopy (adjOR 0.55 [95% CI: 0.31–0.97]) in children. Results were confirmed even including children MDS in the multivariate models.

The cross-sectional study by De Batlle et al. [62] was conducted in Mexico on a random sample of 1476 children. Maternal adherence to the MD during pregnancy was assessed using a validated semi-quantitative 70-item FFQ self-administered at the children age of 6–7 years [80]. High adherence to MD, calculated according to the score of Trichopoulou [40], was associated to reduced risk of current sneezing (OR 0.71 [95% CI: 0.53–0.97]) in children but no associations were found for other endpoints. However, results of this study should be interpreted cautiously because of the reliability of maternal dietary recall after more than 6 years.

Other studies surveyed the offspring at earlier times: 3 studies at 12–18 months of life [63,65,66], one study at 3 years [67], and one study at 4 years [69]. In all of them the outcome was asthma/wheezing; 3 studies included also atopy/atopic eczema [65,67,69] and another study included also rhinitis [69]. Castro-Rodriguez et al. [63] performed an observational study on a Spanish cohort of 1409 infants aged 15–18 months. When children came to receive vaccination at 15–18 months of age, parents were asked to complete a questionnaire emphasizing on wheezing during the first year of life and also on epidemiological and risk/protective factors. At the same time a self-administered semi-quantitative 11-items FFQ were administered to mothers to collect data on their food intake during pregnancy. Maternal adherence to the MD, measured according to the score by Psaltopoulou [64], and consumption of olive oil were both significantly associated with less wheezing in children, but association was only confirmed for olive oil consumption after multivariate analysis. Similarly, other studies did not evidence any association between maternal adherence to the MD and development of wheeze or eczema in the first 15 months of life [65,66]; wheeze, asthma or allergy at 3 years of age [67]; wheezing, rhinitis and dermatitis in the children at 4 years of age [69]. Details of these studies are reported in Table 1.

#### 3.4.6. Body Weight and Metabolic Markers

Evidence for these outcomes comes from 4 cohort studies (2 good, 1 intermediate quality and 1 of poor quality), 2 cross sectional studies of intermediate quality. Cohort studies reached the best scores for quality.

The quality of the diet during the first trimester of pregnancy and its relation to insulin sensitivity/resistance in the new-borns was evaluated in a cross-sectional study involving 35 women [70] who completed a 169-items FFQ 3–5 weeks after delivery. The FFQ was administered by trained dietician, and adherence to the MD was assessed by the healthy eating index (HEI) adapted for the Spanish population [71] and by a modified MDA scores used in the PREDIMED study [72]. Women with low HEI- or MDA scores delivered infants with high insulinaemia (p 0.048 and p 0.017, respectively), homeostatic model assessment for insulin resistance (HOMA-IR) (p 0.031 and p 0.049, respectively) and glycaemia (p 0.018 and p 0.048, respectively). The relative risk (RR) of high-neonatal glycaemia and insulinaemia was 7.6 (p 0.008) and 6.7 (p 0.017) for low vs. high HEI-score groups. High HOMA-IR and high glucose RR were, respectively, 3.4 (p 0.043) and 3.9 (p 0.016) in neonates from the <7 MDA- vs. >7 MDA-score group. These RRs were not affected by potential confounders.

Chatzi et al. [73] evaluated the association between maternal adherence to the MD in early pregnancy and offspring obesity and cardiometabolic risk in two cohorts with different socio-economic characteristics and different geographic locations. Project Viva, a prospective mother–child cohort began in Massachusetts, USA in 1999 (997 mother-child pairs), while the RHEA study, a population-based mother-child cohort study, started in Crete, Greece in 2007 (569 mother-child pairs). In Project Viva, at study enrolment (median 9.9 weeks’ gestation) mothers reported their diet since the time of their last menstrual period using a validated semi-quantitative FFQ. RHEA participants completed a validated FFQ at mean 14.6 weeks gestation. Overall dietary pattern was examined using the Trichopoulou score [40]. Different parameters were evaluated in the children in mid-childhood (median 7.7 years, Viva project) and in early childhood (median 4.2 years, RHEA project). After pooling analysis calculated using mixed models, including cohort and child age at outcome assessment as random effects and all other covariates as fixed effects, for each 3-point increment in the MD score, ranged from 0 (minimal adherence to the MD) to 9 (maximal adherence), offspring BMI z-score was lower by 0.14 units (95% CI: −0.15 to −0.13), waist circumference by 0.39 cm (95% CI: −0.64 to −0.14), and the sum of skin-fold thicknesses by 0.63 mm (95% CI: −0.98 to −0.28). The Authors also observed a lower systolic (−1.03 mmHg [95% CI: −1.65 to −0.42]) and diastolic blood pressure (−0.57 mmHg [95% CI: −0.98 to −0.16]) in offspring.

Fernández-Barrés et al. [74] analysed 1827 mother–child pairs from the Spanish “Infancia y Medio Ambiente” cohort study, recruited between 2003 and 2008, to evaluate associations between adherence to the MD during pregnancy and childhood overweight and abdominal obesity risk. A validated 101-items FFQ was administered to evaluate dietary habits from first to third trimester of pregnancy, and the Mediterranean diet (rMD) score [75] was calculated. No association was evidenced between rMD score and body mass index z-score of children at 4 years of age, whereas there was a significant association between higher maternal adherence to the MD and lower waist circumference in children (−0.62 cm [95% CI: −1.1 to −0.14).

In a cross-sectional study, Gesteiro et al. [76] analysed the MD adherence in 35 Spanish women during the first trimester of pregnancy, who completed 169-items FFQ guided by a trained dietician. Adherence to the MD was calculated with a modified version of the score used in the PREDIMED study [72]. At birth, neonates from mothers with the lower MD score (<7) showed higher cord blood LDL-c (p 0.049), Apo B (p 0.040), Apo A1/Apo B ratio (p 0.024) and increased homocysteine levels (p 0.026).

In a cohort study, Mantzoros and colleagues [77] evaluated the relation between maternal adherence to the MD during pregnancy and cord blood adiponectin and/or leptin level, which have been associated with post-natal body size and adiposity in the first years of life, in 780 American women. Maternal diet assessment at both the first and second trimester was performed using a semi-quantitative FFQ slightly modified for use in pregnancy and MD score calculated accordingly to Trichopoulou [40]. Closer adherence to a MD during pregnancy was not associated with cord blood leptin or adiponectin (p 0.38 and p 0.93, respectively).

Gonzalez-Naqhm et al. [78] evaluated in 390 women enrolled in the Newborn Epigenetic Study the relation between MD adherence, assessed by a 150 items FFQ at preconception or at first trimester, and DNA methylation in cord blood leukocytes at birth. Infants of mothers with low adherence to the MD had greater odds of hypomethylation of the MEG3-IG region (OR 2.80 [95% CI: 1.35–5.82]). Sex-stratified models showed that this association was present in girls only. The MEG3-IG region may be an upstream regulator of the MEG3 DMR, which has been associated with type 2 diabetes [78]. Hypomethylation of MEG3- IG region could explain the association between MD and improvements of type 2 diabetes [81].

## 4. Discussion and Conclusions

In the present review, we systematically summarized studies carried out to verify the protective effect on the offspring of maternal adherence to the MD during pregnancy. All but one of the 29 studies included in the review were observational (cross-sectional, cohort, and case–control) studies.

Although maternal nutrition has been recognized as one of the most important environmental factors influencing foetal growth and development [14,15,16,17], we found intermediate evidence linking maternal adherence to the MD pattern to FGR and risk of SGA new-borns, with cohort studies that reached the best scores for quality (Table 2). A confirmation that adherence to the MD during pregnancy could represent a strategy for reducing the incidence of FGR and SGA new-borns could come from a randomized controlled trial that is recruiting patients in Spain [82]. This study is randomizing women at high risk for growth restricted foetuses into two different behavioural strategies program: a stress reduction program based on mindfulness techniques or a nutrition interventional program based on MD. The trial will last till February 2021.

We founded intermediate evidence supporting a protective effect of MD on preterm delivery, with cohort studies reaching the best scores for quality. The MD pattern, including low amount of sugars, could result in better blood glucose regulation during pregnancy. Although glucose intolerance is associated with a shorter duration of gestation independently of other known risk factors for prematurity [83], not all studies investigating the effect of MDA during pregnancy on preterm delivery found no significant correlation (Table 1). The heterogeneity of results could be in part explained by differences in the definition of preterm delivery (<37 weeks versus earlier periods). Of note, the only randomized controlled trial found in the literature [49] showed that an early nutritional intervention with MD supplemented with extra virgin olive oil and pistachios significantly reduces the rate of preterm delivery.

Since long ago, many studies demonstrated that appropriate intake of folate during pregnancy can prevent the recurrence of NTDs [84]. Although it is known that the MD provides appropriate amount of folate, to date only two cross sectional studies [57,58] have investigated the effects of maternal adherence to the MD on NTDs incidence in offspring. Maternal MD adherence and risk of NTDs appeared significantly related, but the low number of studies reduces the level of evidence. In addition, it is not clear whether the MD protective effect is simply due to the correct folic acid intake or other MD components contribute as well. Anyway, to improve adherence to the MD in the periconception period could be considered a good strategy to reduce the incidence of NTDs. Regarding CHDs and gastroschisis, the number of studies is too low to draw any conclusion.

At present, there is low evidence of a link between maternal adherence to the MD and incidence of asthma and/or allergic diseases in the offspring. This is also due to quality coming from these studies was much more mixed, particularly for cohort studies. Additional studies are needed to better clarify the role of maternal adherence to the MD on this outcome.

Obesity and metabolic syndrome (MetS) are two of the most common chronic diseases among children. Recent evidence suggests these conditions have their roots in utero as maternal obesity, dyslipidaemia, and hyperglycaemia are associated with child cardiometabolic health and developing insulin resistance and obesity later in life [85,86]. Although studies considered in this review indicate that higher adherence to the MD during pregnancy is a potential protective factor against abdominal obesity [73,74] and positively influences lipoprotein and homocysteine concentration [76], and insulin resistance in new-borns [70], the use of different endpoints to evaluate outcomes reduces the level of evidence.

Overall, most of the studies included in this review showed beneficial association between MD adherence during pregnancy and children’s health. The strength of the association varied in the different health outcomes, and the level of evidence was affected by the high heterogeneity among the study design. Heterogeneity was mainly related to the methodology used for assessment of MD adherence. Epidemiological studies commonly use the FFQ to assess usual food consumption. Although an FFQ does not have the same accuracy as a dietary record or a 24 h dietary recall, it can reasonably report intake over a large period [87]. There are many kinds of FFQ, and not all have been validated. Anyway, they differ for the number of food items, the way of administration (self-administration or interviewer-administration), the quantification of consumed foods, etc. Furthermore, there are many ways to analyse results from FFQ, which are often used to extrapolate an index of overall diet quality based on an a priori scoring system [88]. The use of different scores for the assessment of adherence to the MD represents a possible confounding factor while comparing different studies.

In addition, the consumption rate of specific food groups was seldom considered. The so-called MD was inspired by the eating habits of people in the Mediterranean area (mainly Greece, Southern Italy and Spain). MD provides an optimal intake of “positive” nutrients (polyunsaturated fats, fibres, vitamins etc.) and a low intake of “negative” nutrients (e.g., saturated fats, sugars, sodium) through the proportionally high consumption of olive oil, legumes, unrefined cereals, fruits, and vegetables, moderate to high consumption of fish, moderate consumption of dairy products (mostly as cheese and yogurt), moderate wine consumption, and low consumption of non-fish meat products. The proportion of Mediterranean foods in the diet is different in different Mediterranean countries, even in people having the same level of adherence, and it largely depends on the characteristics of the study sample, i.e., ethnic origin of the enrolled population. Culture-driven dietary preferences vary among population and influence the intake of specific food subgroups. These differences are likely incompletely captured in the semiquantitative FFQ, particularly in their shorter version, because of the limited number of food items that are evaluated. Therefore, the sensitivity of MD adherence as predictor of children outcomes could be affected by regional variations of MD.

Another confounding factor is the stage of pregnancy considered for evaluation of adherence to the MD, as well as the time elapsed between the considered period and the administration of the FFQ. Not explored social and environmental factors could have a role.

How a good adherence to the MD during pregnancy could have positive effect in offspring not only during foetal life but also in later life is still unclear. The induction of epigenetics modification represents a possible explanation [78], but further studies are needed to confirm it. In addition to the epigenetics hypothesis, it is known that various nutrients may influence pregnancy outcomes by altering both maternal and foetal metabolism due to their roles in modulating oxidative stress, enzyme function, signal transduction and transcription pathways that occur early in pregnancy.

In conclusion, a good maternal diet quality in general, and the adherence to the MD in particular, are associated with a reduced occurrence of some negative outcomes in babies. Although it is still unclear whether an intervention to promote the MD could effectively reduce the prevalence of some childhood diseases, and randomized control trials are needed to better clarify it, current preconception care recommendations should carefully consider the benefit of MD, reinforcing advice on correct dietary habits. Strategies aiming to promote adherence to MD dietary pattern may be of considerable importance to public health. In addition, they have low cost and no side effects.

## Figures and Tables

**Table 1:** Summary of the included studies.

Author	Geographic Area	Study Design	Included Participants	Assessment of Dietary Habits	Assessment of Adherence to MD	Outcomes	Results
Timmermans et al. (2012) [38]	Netherlands	Prospective population-based cohort study	3207 mothers with a spontaneously conceived live-born singleton pregnancy	Semi-quantitative FFQ (293 items) self-administered during early pregnancy (GA < 18 weeks)	Logistic regression analysis was used to identify a comparable dietary pattern, which was labeled MD as it was characterized by higher intakes of pasta, rice, vegetable oils, fish, vegetables and alcohol, and lower intakes of meat, potatoes and fatty sauces. All women were categorized into equal tertiles based on their probability score for the diet, namely: low MDA, medium MDA and high MDA.	Fetal growth	Low MDA resulted associated with lower birth weight (difference in grams at birth −72 [95% CI: −110.8 to 33.3])
Chatzi et al. (2012) [39]	Spain (INMA cohort) and Greece (RHEA cohort)	Prospective population-based cohort study	Spain: 2461 mother-newborn pairs. Greece: 889 mother-newborn pairs	Semi-quantitative FFQ (100 items in IMNA cohort and 250 items in RHEA cohort) administered by trained interviewers during first (IMNA cohort) or mid trimester (RHEA cohort) of pregnancy	Trichopoulou’s score [40] modified for pregnancy	Fetal growth	High MDA was associated with lower risk of delivering a FGR infant (OR 0.5 [95% CI: 0.3–0.9]) in the INMA-Mediterranean cohort. In all cohort high MD adherence increased birth weight in smoking mothers
Sauders et al. (2014) [41]	Guadeloupe (French Caribbean Island)	Prospective mother-child cohort study	728 pregnant women with a live-born singleton pregnancy without major congenital malformations	Semi-quantitative FFQ (214 items) administered by trained interviewers in the days after delivery	Trichopoulou’s score [40]	Fetal growth and prematurity	No overall associations with FGR. No overall association with prematurity. Decreased risk in overweight and obese woman (adjOR 0.7 [95% CI: 0.6–0.9])
Gomez-Roig et al. (2017) [42]	Spain	Cross-sectional study	46 mothers with SGA fetuses 81 mothers with appropriate for gestational age (AGA) fetuses	Semi-quantitative FFQ (127 items) administered by trained interviewers during the third trimester of pregnancy	Trichopoulou’s score [40]	SGA infants	High MD score was associated with a lower risk of SGA (OR 0.18 [95% CI: 0.74–0.501]) for the third consumer quartile
Peraita-Costa et al. (2018) [43]	Spain	Cross-sectional population-based study	492 mothers	Semi-quantitative FFQ (16 items) self-administered after delivery	Modified KidMed score [44]	SGA infants	The newborns born to women with low MDA presented a higher risk of being SGA (adjOR 1.68 [95% CI: 1.02–5.46]) when adjusting for parental BMI and multiple gestation, but not when adjusting for all significant possible confounders.
Parlapani et al. (2017) [45]	Greece	Single-center, prospective, observational cohort study	82 women who delivered preterm singletons at post conceptional age < 34 weeks	Semi-quantitative FFQ (156 items) self-administered immediately before or after delivery	Panagiotakos dietary score [46]	Fetal growth and prematurity-associated complications	Low MD adherence increased the risk of IUGR, low birth weight, bronchopulmonary dysplasia and necrotizing enterocolitis in preterm infants (<34 weeks)
Martinez-Galiano et al. (2018) [47]	Spain	Prospective multicenter matched case-control study (matching criterion: maternal age at delivery)	518 mothers of singleton SGA infants 518 mothers of singleton infants with normal weight for GA	Semi-quantitative FFQ (137 items) administered by trained interviewers within 2 days after delivery	PREDIMED score [48], Trichopoulou’s score [40], Panagiotakos’ score [46]	SGA infants	MDA and daily consumption of 5 gr of olive oil was associated to a reduced risk of SGA in newborns (adjOR 0.59 [95% CI: 0.38–0.98])
Assaf-Balut et al. (2017) [49]	Spain	Prospective randomized controlled intervention trial	500 mothers allocated to intervention (MD diet supplemented with extra virgin olive oil and pistachios) and 500 allocated to control (standard diet with limited fat intake)	Semi-quantitative FFQ (14 items) administered by trained interviewed during 4 study visits (at first ultrasound visit at 24–28 GA, at 36–38 GA, and at delivery) to evaluate the adherence to the intervention	MDA screener score [50]	SGA infants and prematurity	MD supplemented with extravergin olive oil and pistachios significantly reduced prematurity rate (p 0.023) and SGA (p 0.001) in the intervention group
Carmichael et al. (2013) [51]	United States	Cross-sectional study	5738 mothers with a singleton pregnancy who delivered non-malformed infants	Semi-quantitative FFQ (58 items) administered by telephone interview 6 weeks—24 months after delivery	Trichopoulou’s score [40] and DQI incorporating pregnancy-specific nutritional recommendations [52]	Prematurity	No association
Mikkelse et al. (2008) [53]	Denmark	Prospective cohort study	35657 pregnant women with a live-born singleton pregnancy	Semi-quantitative FFQ (360 items) self-administered at mid-pregnancy (week 25) by mail	Khoury’s score [54]	Prematurity	High MDA reduced the risk of early preterm birth (adjOR 0.28 [95% CI: 0.11–0.76]). No associations with late preterm delivery.
Haugen et al. (2008) [55]	Norway	Prospective cohort study	26563 pregnant women with a live-born singleton pregnancy	Semi-quantitative FFQ (255 items) self-administered at week 18–22 of pregnancy	Khoury’s score [54]	Prematurity	No association
Smith et al. (2015) [56]	United Kingdom	Population-based cohort study	922 mothers with singleton late and moderate preterm (LMPT) births 965 mothers with singleton term births	Maternal interview shortly after delivery	MDA on the basis of the presence of at least 1 of the following major criteria: five portions of fruit and vegetables every day; fish more than twice a week; meat no more than twice a week; max two cups of coffee/d.	Late and moderately preterm (LMPT) birth	Higher risk of delivering LMPT in not adherent women (RR 1.81 [95% CI: 1.04–3.14])
Vujkovic et al. (2009) [57]	Netherlands	Retrospective multicenter case-control study	50 mothers of children with spina bifida 81 control mothers	Semi-quantitative FFQ (200 items) administered 14 months after delivery and individually checked for consistency at the hospital or by telephone by the researcher.	Principal component factor analysis (PCA) and reduce rank regression (RRR) were used to identify a comparable dietary pattern, which was labeled MD as it was characterized by high intake of vegetables, fruits, vegetable oils, legumes, fish, alcohol and cereal products and low intakes of potatoes and sweets.	NTDs	Low MDA according to both PCA and RRR, was associated with an increased risk of spina bifida (OR 2.7 [95% CI: 1.2–6.1] and OR 3.5 [95% CI: 1.5–7.9], respectively)
Carmicheal et al. (2012) [58]	United States	Retrospective multicenter case-control study	936 mothers of children with NTDs 2475 mothers of children with orofacial clefts 6147 control mothers	Semi-quantitative FFQ (58 items) administered by telephone interviews 6 weeks–24 months after delivery	Trichopoulou’s score [40] and DQI [52] incorporating pregnancy-specific nutritional recommendations	NTDs and orofacial clefts	High Trichopoulou score and DQI score were protective for NTDs, with a stronger association observed for anencephaly (adjOR 0.64 [95% CI: 0.45–0.92] and 0.49 [95% CI: 0.31–0.75], respectively)
Botto et al. (2016) [59]	USA	Population based, multicenter case-control study	9885 case mothers 9468 control mothers	Semi-quantitative FFQ (58 items) administered by telephone interviews 6 weeks–24 months after delivery	Trichopoulou’s score [40] and the DQI [52] incorporating pregnancy-specific nutritional recommendations	Congenital Heart Defects	High Trichopoulou’s score was protective only for perimembranous ventricular septal defects (14%, OR 0.86 [95% CI: 0.69–1.07]). High DQI was protective for tetralogy of Fallot (OR 0.63 [95% CI: 0.49–0.80]), conotruncal defects (OR 0.76 [95% CI: 0.64–0.91]), atrial septal defects (OR 0.77 [95% CI: 0.63–0.94]) and for all septal defects (OR 0.86 [95% CI: 0.75–1.00]).
Feldkamp et al. (2014) [60]	USA	Population based, multicenter case-control study	1125 gastroschisis cases 9483 controls	58-item FFQ (58 items) administered by a computerized-assisted telephone interview (CATI) to case and control mothers 6wk to 24 months delivery	Trichopoulou’s score [40] and the DQI [52] incorporating pregnancy-specific nutritional recommendations	Gastroschisis	High Trichopoulou’s score (quartile 2, adjOR0.62 [95% CI: 0.33–1.16]; quartile 3, adjOR0.51 [95% CI: 0.28–0.94]; quartile 4, adjOR0.50 [95% CI: 0.28, 0.90]) and DQI score (quartile 2, adjOR 0.58 [95% CI: 0.40–0.86]; quartile 3, adjOR 0.52 [95% CI: 0.36–0.79]; quartile 4, adjOR 0.48 [95% CI: 0.32–0.76]) were protective for gastroschisis.
Chatzi et al. (2008) [61]	Spain	Cohort study	460 children	Semi-quantitative FFQ (42 items) referred to the pregnancy and administered to mothers 3 months after delivery by a face-to-face interview. Semi-quantitative FFQ (96 items) administered to the parents of the children at 6.5 year of age by an interviewer	Trichopoulou’s score [40]	Wheeze, atopic wheeze and atopy at 6.5 years	High MDS in mothers was protective for persistent wheeze (adjOR 0.22 [95% CI:0.08–0.58]), atopic wheeze (adjOR0.30 [95% CI:0.10–0.90]), and atopy (adjOR 0.55 [95% CI: 0.31–0.97]) in children at 6,5 years
De Batlle et al. (2008) [62]	Mexico	Cross-sectional study	1476 children	Semi-quantitative FFQ (70 items) referred to the pregnancy and self-administered at the children age of 6–7 years	Trichopoulou’s score [40]	Asthma, Wheezing, rhinitis, sneezing, itchy-watery eyes at 6-7 years	High MDS was protective for current sneezing (OR 0.71 [95% CI: 0.53–0.97]).
Castro-Rodriguez et al. (2010) [63]	Spain	Cohort study	1409 infants	Semi-quantitative FFQ (11 items) referred to the pregnancy and self-administered at the children’s aged of 15–18 months	MDS modified from Psaltopoulou [64]	Wheeze at 12 months	MD (p 0.036) and olive oil (p 0.002) were associated with less wheezing. Only olive oil intake remained inversely associated with wheezing (adjOR 0.57 [95% CI: 0.4–0.9])
Chatzi et al. (2013) [65]	Spain (INMA cohort) and Greece (RHEA cohort)	Cohort study	Spain: 1771 mother-newborn pairs. Greece: 745 mother-newborn pairs	Semi-quantitative FFQ (100 items in IMNA cohort and 250 items in RHEA cohort) administered by trained interviewers at mean 13.8 weeks of GA (IMNA cohort) or 14.6 weeks of GA (RHEA cohort)	Trichopoulou’s score [40] modified for pregnancy considering dairy food protective and not including in the score alcohol consumption.	Wheeze and eczema at 12 months	No associations between MD score and wheeze and eczema
Alvarez-Zallo et al. (2018) [66]	Spain	Cohort study	1087 mother-infant pairs	Semi-quantitative FFQ (11-items) referred to the pregnancy and self-administered at the children aged 12–15 months	MDS modified from Psaltopoulou [64]	Wheeze and eczema at 12–15 months	No associations between MD score and wheezing, recurrent wheezing and eczema
Lange et al. (2010) [67]	United States	Cohort study	1376 mother-infant pairs	Semi-quantitative FFQ (166 items) self-administered at the first and second trimesters visits	MD score modified from Trichopoulou [40], Alternate Healthy Eating Index modified for pregnancy [68] and PCA to look at Western and Prudent diets	Wheeze, asthma and atopy at 3 years	No associations between dietary patterns and asthma, atopy or wheezing
Castro-Rodriguez et al. (2016) [69]	Spain	Cohort study	1000 mother-newborn pairs	Semi-quantitative FFQ (11 items) regarding the consumption of foods during pregnancy self-administered at the time point of 1.5 years of children’s life. Semi-quantitative FFQ (11 items) regarding the consumption of food by the child self-administered at the time point of 4 years of life	MDS modified from Psaltopoulou [64]	Wheeze, dermatitis and allergic rhinitis at 4 years	No associations between MD score and wheezing, rhinitis and dermatitis
Gesteiro et al. (2012) [70]	Spain	Cross sectional study	35 women	169 items FFQ conducted by a trained dietician 3–5 after delivery	Healthy eating index (HEI) adapted for the Spanish population [71] and by a modified MDA scores used in the PREDIMED study [72]	Various insulin sensitivity/resistance biomarkers at birth	Low HEI- or low MDA-score diet delivered infants with high insulinaemia (p 0.048 or p 0.017, respectively), HOMA-IR (p 0.031 or p 0.049, respectively) and glycaemia (p 0.018 or p 0.048, respectively). The relative risk (RR) of high-neonatal glycaemia and insulinaemia were 7.6 (p 0.008) and 6.7 (p 0.017) for low vs. high HEI-score groups. High HOMA-IR and high glucose RR were, respectively, 3.4 (p 0.043) and 3.9 (p 0.016) in neonates from the <7 MDA- vs. >7 MDA-score group.
Chatzi et al. (2017) [73]	USA (Project Viva cohort) and Greece (RHEA cohort)	Prospective mother–child cohort study	997 mother–child pairs from Project Viva and 569 pairs from the RHEA study	In Project Viva, mothers reported their diet since the time of their last menstrual period at study enrolment (median 9.9 weeks gestation) using a validated semi-quantitative FFQ. RHEA participants completed a validated FFQ at mean 14.6 weeks gestation.	Trichopoulou’s score [40]	BMI z-score, waist circumference, skin-fold thickness, systolic and diastolic blood pressure	In the pooled analysis, for each 3-point increment in the MDS, offspring BMI z-score was lower by 0.14 units (95% CI: −0.15 to −0.13), waist circumference by 0.39cm (95% CI: −0.64 to −0.14), the sum of skin-fold thicknesses by 0.63mm (95% CI: 0.98 to −0.28), systolic blood pressure by −1.03 mmHg (95% CI: −1.65 to −0.42) and diastolic blood pressure by −0.57 mmHg (95% CI: −0.98 to −0.16).
Fernández-Barrés et al. (2016) [74]	Spain	Population based cohort study	1827 pairs of mother and children	Validated 101 items FFQ conducted from first to third trimester	RelativeMediterranean diet score (rMED) [75]	BMI and waist circumference	A significant association between higher adherence to MD and lower waist circumference (−0.62 cm [95% CI: −1.1 to −0.14]).
Gesteiro et al. (2015) [76]	Spain	Cross sectional study	35 women	Complete 169 items FFQ guided by a trained dietician conducted at first trimester	Modified MDA scores used in the PREDIMED study [72]	Cord blood lipoprotein and homocysteine concentrations	Mothers at the low MDA-score delivered neonates with high cord blood LDL-c (p 0.049), Apo B (p 0.040), homocysteine (p 0.026) and Apo A1/Apo B ratio (p 0.024).
Mantzoros et al. (2010) [77]	USA	Prospective cohort study	780 women	Slightly modified semi-quantitative FFQ at both the first and second trimester	Trichopoulou’s score [40]	Cord blood leptin and adiponectin concentrations	Closer adherence to a Mediterranean pattern diet during pregnancy was not associated with cord blood leptin (p 0.38) or adiponectin (p 0.93)
Gonzalez-Nahm et al. (2017) [78]	USA	Cohort study	390 women whose infants had DNA methylation data available from cord blood leukocytes	150 items FFQ at preconception or at first trimester	Modified Trichopoulou’s score [40]	Methylation at the MEG3-IG region	Infants of mothers with a low adherence to a Mediterranean diet had a greater odd of hypo-methylation at the MEG3-IG differentially methylated region (OR 2.80 [95% CI: 1.35−5.82])

adjOR: adjusted odds ratio; BMI: body mass index; CI: confidence interval; GA: gestational age; FFQ: food frequency questionnaire; MEG3-IG: maternally expressed gene 3 - intergenic region; MD: Mediterranean Diet; MDA: Mediterranean Diet adherence; NTDs: neural tube defects; OR odds ratio; PTD: preterm delivery; RR: relative risk; SGA: small for gestational age; LMPT: Late and moderately preterm; HOMA-IR: homeostatic model assessment for insulin resistance.

**Table 2:** Risk of bias of the included studies.

Author	Study Type	Tool for Assessment	Quality
Timmermans et al. (2012) [38]	Cohort	STROBE	24/33—Intermediate
Chatzi et al. (2012) [39]	Cohort	STROBE	27/33—Good
Sauders et al. (2014) [41]	Cohort	STROBE	24/33—Intermediate
Gomez-Roig et al. (2017) [42]	Cross Sectional	STROBE	12/33—Poor
Peraita-Costa et al. (2018) [43]	Cross Sectional	STROBE	16/33—Intermediate
Parlapani et al. (2017) [45]	Cohort	STROBE	21/33—Intermediate
Martinez-Galiano et al. (2018) [47]	Case-Control	STROBE	22/33—Intermediate
Assaf-Balut et al. (2017) [49]	RCT	Cochrane ROB Tool	Poor quality due to blindness and allocation concealment
Carmichael et al. (2013) [51]	Cross Sectional	STROBE	11/33—Poor
Mikkelsen et al. (2008) [53]	Cohort	STROBE	19/33—Intermediate
Haugen et al. (2008) [55]	Cohort	STROBE	22/33—Intermediate
Smith et al. (2015) [56]	Cohort	STROBE	26/33—Good
Vujkovic et al. (2009) [57]	Case-Control	STROBE	21/33—Intermediate
Carmicheal et al. (2012) [58]	Case-Control	STROBE	17/33—Intermediate
Botto et al. (2016) [59]	Case-Control	STROBE	18/33—Intermediate
Feldkamp et al. (2014) [60]	Case-Control	STROBE	23/33—Intermediate
Chatzi et al. (2008) [61]	Cohort	STROBE	25/33—Intermediate
De Batlle et al. (2008) [62]	Cross Sectional	STROBE	12/33—Poor
Castro-Rodriguez et al. (2010) [63]	Cohort	STROBE	26/33—Good
Chatzi et al. (2013) [65]	Cohort	STROBE	28/33—Good
Alvarez-Zallo et al. (2018) [66]	Cohort	STROBE	14/33—Poor
Lange et al. (2010) [67]	Cohort	STROBE	31/33—Good
Castro-Rodriguez et al. (2016) [69]	Cross Sectional	STROBE	18/33—Intermediate
Gesteiro et al. (2012) [70]	Cross Sectional	STROBE	16/33—Intermediate
Chatzi et al. (2017) [73]	Cohort	STROBE	26/33—Good
Fernández-Barrés et al. (2016) [74]	Cohort	STROBE	27/33—Good
Gesteiro et al. (2015) [76]	Cross Sectional	STROBE	22/33—Intermediate
Mantzoros et al. (2010) [77]	Cohort	STROBE	25/33—Intermediate
Gonzalez-Nahm et al. (2017) [78]	Cohort	STROBE	13/33—Poor
